# Integration of HIV/AIDS services into African primary health care: lessons learned for health system strengthening in Mozambique - a case study

**DOI:** 10.1186/1758-2652-13-3

**Published:** 2010-01-20

**Authors:** James Pfeiffer, Pablo Montoya, Alberto J Baptista, Marina Karagianis, Marilia de Morais Pugas, Mark Micek, Wendy Johnson, Kenneth Sherr, Sarah Gimbel, Shelagh Baird, Barrot Lambdin, Stephen Gloyd

**Affiliations:** 1 University of Washington Department of Global Health, Harborview Medical Center, 325 9th Ave, Box 359931, Seattle, WA 98104, USA; 2 Health Alliance International, 4534 11th Ave NE, Seattle, WA 98105, USA; 3 Mozambique Ministry of Health Ministério da Saúde C.P. 264 Av. Eduardo Mondlane/Salvador Allende, Maputo, Republica de Moçambique; 4 Provincial Health Directorate, Sofala Province, Ministério da Saúde C.P. 264 Av. Eduardo Mondlane/Salvador Allende, Maputo, Republica de Moçambique; 5 Provincial Health Directorate, Manica Province, Ministério da Saúde C.P. 264 Av. Eduardo Mondlane/Salvador Allende, Maputo, Republica de Moçambique

## Abstract

**Introduction:**

In 2004, Mozambique, supported by large increases in international disease-specific funding, initiated a national rapid scale-up of antiretroviral treatment (ART) and HIV care through a vertical "Day Hospital" approach. Though this model showed substantial increases in people receiving treatment, it diverted scarce resources away from the primary health care (PHC) system. In 2005, the Ministry of Health (MOH) began an effort to use HIV/AIDS treatment and care resources as a means to strengthen their PHC system. The MOH worked closely with a number of NGOs to integrate HIV programs more effectively into existing public-sector PHC services.

**Case Description:**

In 2005, the Ministry of Health and Health Alliance International initiated an effort in two provinces to integrate ART into the existing primary health care system through health units distributed across 23 districts. Integration included: a) placing ART services in existing units; b) retraining existing workers; c) strengthening laboratories, testing, and referral linkages; e) expanding testing in TB wards; f) integrating HIV and antenatal services; and g) improving district-level management. Discussion: By 2008, treatment was available in nearly 67 health facilities in 23 districts. Nearly 30,000 adults were on ART. Over 80,000 enrolled in the HIV/AIDS program. Loss to follow-up from antenatal and TB testing to ART services has declined from 70% to less than 10% in many integrated sites. Average time from HIV testing to ART initiation is significantly faster and adherence to ART is better in smaller peripheral clinics than in vertical day hospitals. Integration has also improved other non-HIV aspects of primary health care.

**Conclusion:**

The integration approach enables the public sector PHC system to test more patients for HIV, place more patients on ART more quickly and efficiently, reduce loss-to-follow-up, and achieve greater geographic HIV care coverage compared to the vertical model. Through the integration process, HIV resources have been used to rehabilitate PHC infrastructure (including laboratories and pharmacies), strengthen supervision, fill workforce gaps, and improve patient flow between services and facilities in ways that can benefit all programs. Using aid resources to integrate and better link HIV care with existing services can strengthen wider PHC systems.

## Introduction

The rapid scale up of antiretroviral treatment (ART) and HIV care across Africa over the past five years has provoked an important and lively debate about the impact of "vertical" disease-specific programming on primary health care (PHC) services [1-4]. The major increases in international funding designated for HIV/AIDS programmes from the US President's Emergency Plan for AIDS Relief, the Global Fund to Fight AIDS, Tuberculosis and Malaria, and a range of other donors has raised concerns about how the new funding intersects with existing services.

Most worrisome are charges that HIV/AIDS efforts may distract attention and shift scarce resources away from other urgent health priorities, such as tuberculosis (TB), malaria, diarrheal disease, acute respiratory illness, and immunization [1,2,5]. On the other hand, some have argued that the new large-scale funding for HIV offers an opportunity to rebuild dilapidated health systems. The attention focused on HIV provides a rare opening to harness major funding for health system strengthening [6,7]. Recent proponents of a "diagonal" approach to global health funding similarly argue that disease-specific funding should be used for wider health system strengthening [8,9].

It is still common in Africa to see newly constructed, well-staffed HIV clinics side by side with crumbling PHC facilities, with little integration and few linkages between services. Donor pressure to place large numbers of people on ART as quickly as possible has often subordinated broader population health needs and the health system requirements necessary to address them. In some cases, parallel logistics and delivery systems have been established in order to ensure rapid scale up, leading to imbalances in resource allocation with potentially harmful long-term consequences for other health services [10].

Of equal importance, practitioners on the ground increasingly recognize that quality HIV care cannot be provided without improvements in TB, antenatal, malaria, outpatient and inpatient care services, and basic administrative systems [11-15]. The Mozambique experience with the integration of HIV care services into its public sector PHC system, described in this paper, provides evidence that a "diagonal" implementation strategy can simultaneously strengthen both HIV/AIDS services and the broader health system in which those services are embedded.

### Primary health care in Mozambique

Soon after independence in 1975, Mozambique embraced the comprehensive Alma Ata PHC model [16,17]. The new public sector system provided basic services through a tiered network of linked hospitals, health centres and health posts coordinated through 10 provincial health directorates. The PHC system was undermined by a decades-long war supported by the apartheid governments of Rhodesia and South Africa, followed by severe government spending cutbacks imposed by an International Monetary Fund-led structural adjustment programme [17,18].

HIV/AIDS prevention and treatment services were grafted onto this struggling system when voluntary counselling and testing (VCT) and prevention of mother to child transmission (PMTCT) were initiated in 2001 and the ART scale up began in mid-2004. Much of the new aid funding would initially flow to non-governmental organizations (NGOs) rather than the public system, further reinforcing vertical approaches to treatment expansion.

### ART scale up and integration

After rapidly initiating ART scale up through a vertical "day hospital" approach, the Mozambique Ministry of Health (MOH) recognized the model's limitations and initiated a systematic effort in 2005 to decentralize HIV programmes to sites across the provinces through the existing public sector PHC network. Decentralization required the integration of HIV-related programmes into PHC services to maximize efficient use of the ministry's extremely limited resources. This paper describes the MOH integration process, as supported by a US-based NGO, Health Alliance International, in the central provinces of Manica and Sofala, where HIV prevalence rates of 18% and 23%, respectively, are among the highest in the country [19].

For the purposes of this paper, integration refers to: (1) co-location of different services within the same facility, even if those specific services remain separately staffed; (2) training of personnel to provide multiple services; (3) provision of tools, processes and training to better link separate services; (4) strengthening of linkages, referral and follow up between facility levels; and (5) harmonization of logistics systems, such as data collection, drug and material distribution, transport and supervision across services.

Through the integration process, HIV resources have been used to rehabilitate PHC infrastructure, strengthen supervision, fill workforce gaps and improve patient flow in ways that can benefit all programmes. As a result of integration, the PHC system has been able to test more patients for HIV, place more patients on ART quicker and more efficiently, reduce loss to follow up especially among pregnant women, and achieve greater geographic HIV care coverage compared to the previous vertical model.

## Case description

### 2001-2005: The vertical scale up of HIV/AIDS services

An HIV prevention structure was initiated in 2001. VCT constituted the main element of the approach in Manica and Sofala provinces, and was established as a separate programme with its own freestanding sites and data gathering system. PMTCT services were initiated in 2001 in selected health centres, with parallel data collection and activity duplication for maternal & child health nurses.

The initial approach to ART scale up in 2004 focused on a vertical, donor-initiated, day hospital model in which new freestanding HIV treatment hospitals were constructed in large population centres alongside existing hospital compounds. Day hospitals included their own pharmacies, data systems, health workforce, waiting areas and receptions. Using this separate infrastructure, patients identified as HIV positive from other sectors of the health system (VCT, PMTCT, blood bank and laboratory) were referred to day hospitals to register for HIV care, and to follow a sequence of visits for clinical staging, CD4 testing, social worker visits, treatment for opportunistic infections, and initiation and follow up of ART.

The day hospitals included specifically allocated staff (often expatriate) and better working conditions than other sectors. This vertical approach may have contributed to high loss-to-follow-up rates and missed opportunities that limited the uptake of patients initiating ART. All the data presented in this case description are derived from routine health system data systems. Paper registries are used to collect facility-level data that are later computerized at district level into the MOH health information system. Health Alliance International technical advisors supported data collection, compilation and analysis for programme evaluation to produce findings presented here.

### ART scale up

In Manica and Sofala, the first day hospitals were completed by 2004 in the cities of Chimoio and Beira, respectively. In the first two years, the day hospitals successfully placed nearly 4000 patients on ART. However, providers and planners soon realized that the vertical model had major limitations:

• Day hospitals were only accessible to local urban populations.

• Major loss to follow up (LTFU) at a number of steps in the treatment cascade limited patient uptake. In 2005, only 78% of HIV-positive patients referred to day hospitals returned for CD4 testing, and only 46% of those who returned for results and were found to be ART eligible succeeded in starting treatment.

• Poor linkages with other specific services contributed to LTFU, missed opportunities for testing, and low referral rates.

• Greater human and material resources for HIV-related activities, including salary top-ups, created resentment and limited support from other sectors of the health system.

• Day hospital carrying capacities limited new patient registration.

• Allocation of HIV resources did not strengthen the wider system.

### Voluntary counselling and testing

VCT sites began referrals to vertical day hospitals when ART became available, but distance between facilities contributed to high loss-to-follow-up rates. In 2005, only 59% of those testing positive at VCT sites managed to enrol in HIV care at the day hospitals. CT was not offered in general outpatient and inpatient wards. Doctors could only refer suspected HIV cases to separate freestanding VCT sites. After nearly two years of ART scale up, only 5% of TB patients had been tested before integration efforts began in late 2005. HIV patients were also not being routinely tested and referred to TB care.

### Opportunistic infection identification and management

The initial vertical approach prevented the referral of patients presenting with HIV-related opportunistic infections (OIs) in other service areas because clinicians outside day hospitals were not trained to recognize OIs or make referrals. The missed opportunities were compounded by the lack of provider-initiated CT in outpatient or inpatient services. Even if a clinician did recognize an OI, she had to refer the patient to an off-site VCT centre, where HIV-positive patients would be referred back to the day hospital, creating additional visits and greater loss to follow up.

### Prevention of mother to child transmission

PMTCT was initially established as a prevention activity that focused on single-dose nevirapine distribution coordinated through antenatal care services. When ART became available, HIV-positive mothers were referred to day hospitals for treatment, but in practice they suffered high loss to follow up as few women managed to register. In 2005, only 30% of pregnant women who tested positive in PMTCT programmes enrolled at the day hospitals. By the end of 2005, only 20% of eligible mothers had initiated ART.

### Parallel systems

By 2005, separate data, pharmacy systems, supervision and infrastructure had been set up in two provinces to support the ART scale up. Dozens of new staff, including doctors, nurses, physicians' assistants and administrative staff, had been placed in the day hospitals to focus on ART.

### 2005-2008: Integration and decentralization of HIV/AIDS services

By mid-2005, the MOH recognized these challenges and determined that both decentralization and integration of HIV-related services would be necessary to increase coverage, maximize efficiency and improve quality. Decentralization was necessary to expand coverage to widely distributed populations. Integration would make decentralization possible by maximizing utilization of limited space, infrastructure and health workforce, while improving system efficiency and quality through better service linkages to reduce LTFU and missed opportunities.

### Decentralization and integration of ART into PHC

ART initially expanded into rural hospitals and health centres in areas with higher population densities, while sites were established at smaller centres that would receive regular visits by teams of ART providers. Sites were chosen based on geographic coverage needs, patient volume and assessments of sufficient infrastructure. By December 2008, ART was being provided in 67 sites (of a total 222 health facilities), distributed throughout all 23 districts in the two provinces, where an estimated 360,000 were HIV positive and an estimated 75,000 were ART eligible. Nearly 180,000 people living with HIV/AIDS (PLHIV) registered in the system, and nearly 30,000 had initiated ART out of an estimated 60,000 determined to be ART eligible through CD4 counts.

The actual model of ART provision varies somewhat by site, depending on space, available workforce, and patient volume. In some sites, there are still staff members dedicated for ART, but they are co-located in the same facilities where other key services are provided. In other smaller sites, health centre clinicians were trained in ART provision, which was then integrated with other routine activities, including the regular outpatient consults, inpatient treatment, and ANC. Health Alliance International helped cover "gap year" funding (while the MOH prepared to absorb the new hires) for newly trained nurses and physician's assistants to speed new personnel into the PHC system to help mitigate workforce shortages and support overburdened staff.

### Impact on patient flow and loss to follow up

**Figure 1:**
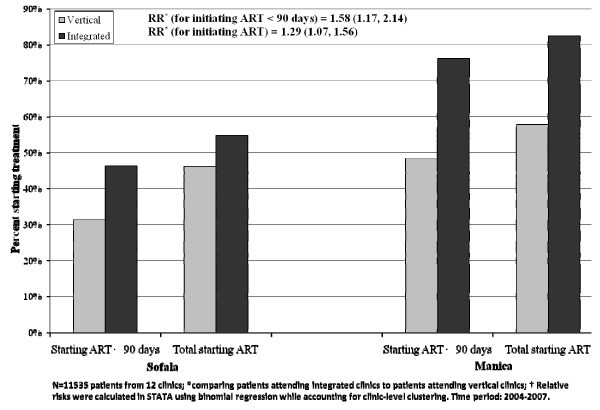
**The percentage of eligible patients starting ART by health facility type and province**.

Analysis of routine data comparing the previous day hospital vertical sites with newer and smaller integrated sites shows that a greater percentage of local PLHIV managed to start ART, and that the percentage of patients initiating ART in less than 90 days (from registration at a health unit) was significantly greater in integrated sites than in vertical day hospitals (see Figure 1). Co-location of ART services with other PHC services in the same facilities reduced LTFU from testing to registration for HIV care, as well as the time from registration to initiation of treatment.

### Monitoring and evaluation, laboratory, pharmacy

Supervision visits for ART are now integrated and conducted with other programme heads. As a result of a national initiative, monitoring and evaluation and other routine data collection tools now integrate ART-related data into the national health information database. Most routine data are still collected through paper-based registries at facility level, but are now computerized at district level and include key ART indicators.

**Figure 2:**
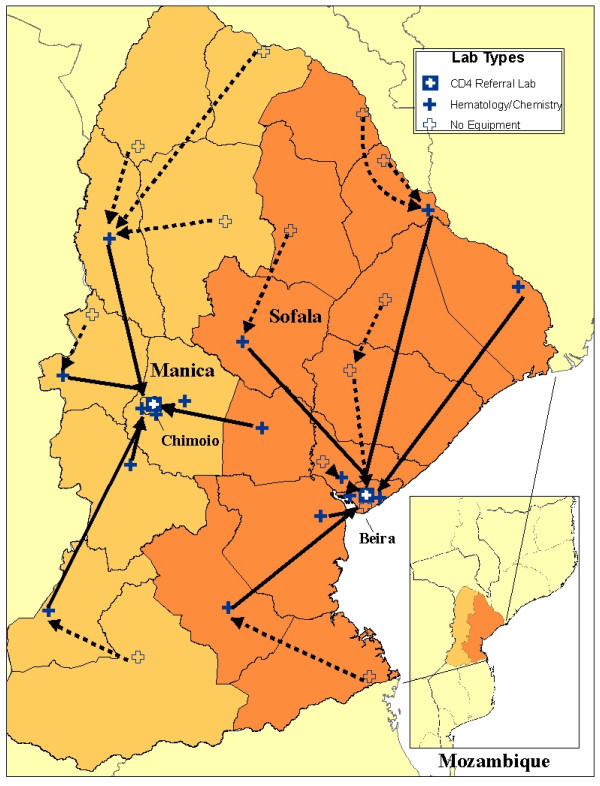
**Province-level PHC laboratory system strengthened using HIV-specific resources**.

The day hospital model did not include separate laboratories, but the MOH-managed provincial lab network has been strengthened via rehabilitation of laboratory facilities, provision of additional equipment, and training to support dispersed sites providing ART services (see Figure 2). National policy mandated that parallel pharmacies in day hospitals be phased out and tasks integrated into existing pharmacies. Provincial and district pharmacists received additional training to integrate the distribution of antiretrovirals (ARVs) into the national system while basic logistics systems were improved to support new ART sites.

### Counselling and testing

**Figure 3:**
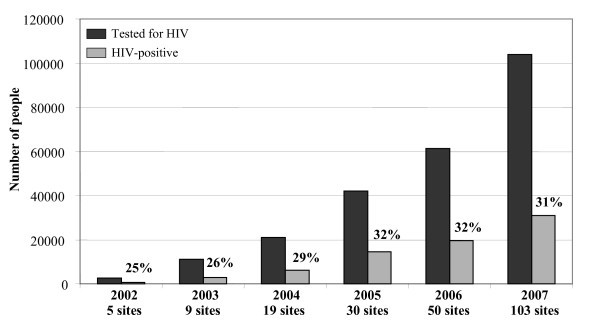
**The number of testing sites and patients tested for HIV (2002-2007)**.

Provider-initiated HIV testing was progressively introduced into other PHC services as health workers were trained in CT. CT is provided routinely in ANC and TB programmes, and offered to patients who present with OIs in other outpatient and inpatient services. Counselling is provided through individual, group or video sessions. In 2004, 20,000 people had been tested in 19 separate VCT sites in both provinces. With integrated CT, more than 100,000 were tested in 103 sites in 2007 alone, including TB services (Figure 3).

**Figure 4:**
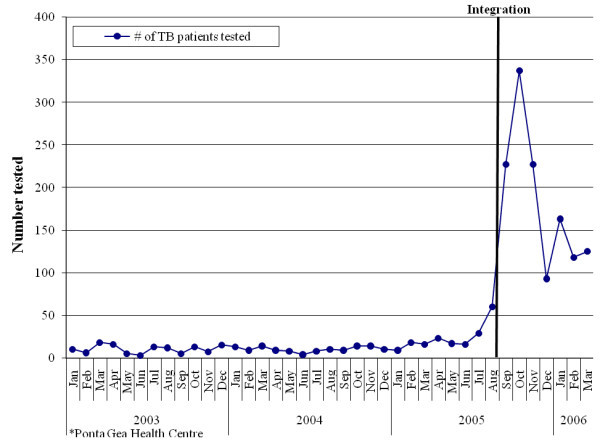
**The number of TB patients tested for HIV per month before/after integration**.

Before these efforts, less than 5% of TB patients were being tested for HIV. With integration efforts, TB staff was trained in CT and new protocols for proper referrals and follow up to HIV care. Items were added to existing patient registries to ensure patient follow up. Clinicians were trained to ensure that HIV patients were also routinely tested for TB and referred, using new protocols and modified paper registries. (See Figure 4 for testing increases in one facility.) By December 2008, more than 90% of TB patients had been tested for HIV at 28 facilities with TB services, and 65% of eligible TB patients had initiated ART.

### Opportunistic infections

By December 2008, 727 doctors, physicians' assistants (called "*tecnicos" *in Mozambique) and nurses had received specific OI training, covering virtually all clinical health workers in the 67 integrated sites. The OI courses integrated closely with CT and referral training. New protocols and modified patients registries were introduced to facilitate referrals and follow up.

### PMTCT

**Figure 5:**
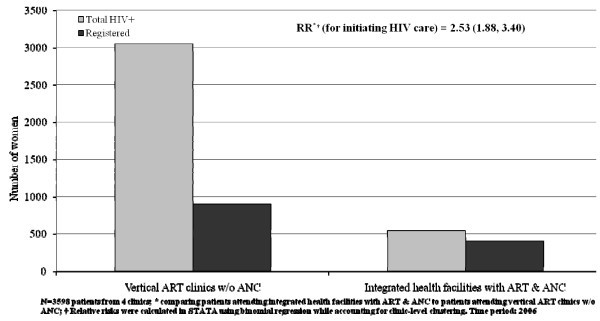
**Number of HIV-positive women referred from PMTCT/ANC and registered for HIV care <30 days post-test**. RR (relative risk) 2.53 for health facility with ANC vs health facility w/o ANC (1.88, 3.40); p < 0.001.

With integration, PMTCT services were included as an integral component of antenatal care (ANC) services within the integrated sites that also initiated ART services. (Routine syphilis testing and treatment, and intermittent preventive therapy for malaria were included in the integrated package.) Maternal & child health nurses and staff were trained in the integrated protocol. Figure 5 compares sites in 2006 offering ART but not ANC (two of the first day hospitals), and sites where both ART and ANC are offered in the same health facility.

Data were gathered for the same time period for both sets of sites. In 2006, the first two larger sites initially remained vertical, while smaller surrounding sites were integrated. The two integrated sites referenced in Figure 5 are similar to the many other integrated sites in the two provinces. Data were gathered for both sets of sites over the same time period. At the large vertical sites, HIV-positive women were referred from other health units with ANC services, while in integrated sites, patients were referred from ANC services within the same health unit.

Loss to follow up from referrals of HIV-positive women from PMTCT services to ART services was reduced from 70% in vertical sites to 25% at integrated sites, based on analysis of routine data. Nearly all integrated sites across both provinces demonstrate similar results. Larger patient volume at the vertical sites may have contributed to higher LTFU, but the dramatic improvement at integrated sites suggests that integrating ANC and ART in the same health units helps reduce LTFU.

### Infrastructure improvements

By the end of 2008, HIV-related resources had contributed to the rehabilitation or construction of 40 staff houses, 22 laboratories, 11 pharmacies and warehouses, and dozens of maternities and ANC service areas. Haematology and biochemistry equipment has been provided for 14 laboratories, and 100 dual system (gas and electricity) refrigerators were purchased for cold chain improvements. Fifty-five motorcycles and 19 cars have been provided to the health system over three years and used for integrated activities. Had a vertical approach been maintained, these funds would have been channelled only to HIV-specific facilities and systems. Table 1 lists the types of infrastructure support provided by HIV-related funding.

**Table 1:** PHC infrastructure improvements supported by HIV-related resources

Infrastructure: buildings	Type	Number
Newly built	Staff residences	40
	
	Waiting areas	2
	
	Warehouse	2

Renovated	Laboratories	22
	
	Pharmacies	11
	
	Outpatient ward	1
	
	Emergency ward	1
	
	Maternity	2

**Infrastructure:** **vehicles, other**	**Type**	**Number**

	4 × 4 vehicles	15
	
	Pick-up trucks	4
	
	Motorcycles	55
	
	Bicycles	621
	
	Refrigerators	34

**Human resources**	**Type**	**Number**

Pre-service training*	Pharmacists	28
	
	Laboratory workers	21
	
	MCH nurses	112

In-service training	HBC workers	679
	
	VCT counsellors	472
	
	PMTCT nurses	870
	
	HIV clinic staff	1465
	
	OI training	727
	
	TB/HIV providers	11

*Participants are required to spend 2-3 years in Manica or Sofala province as a condition of their training.

MCH: Maternal & child health

HBC: Home-based care

## Discussion and evaluation

Decentralization and integration of HIV care services into the existing PHC system in Mozambique has improved: (1) access to care through expansion of sites and services; (2) service quality through reduced LTFU and improved patient flow; and (3) system efficiency by linking services and improving referrals rates, while accelerating the pace at which services can be expanded.

In turn, the integrated approach has channelled HIV/AIDS resources into basic PHC systems, thereby improving overall PHC services. Management of referrals and patient flow will become even more complex and challenging in the near future as patient volume increases. Pressure on basic logistics systems, drug distribution and laboratory facilities will also grow rapidly; the overall health system must be strengthened to meet both short-term and long-term HIV/AIDS treatment goals.

There are, of course, ongoing challenges to successful integration in decentralized sites. Workforce shortages continue to be the single greatest challenge to scale up, and adding HIV care tasks to overburdened staff may raise concerns about quality. Leadership and management training for health directors has been useful to support more efficient human resources management, but further evaluation is necessary to measure quality improvement. It is hoped that integration will bring additional efficiencies overall that can help mitigate the effects of human resource constraints on quality and sustainability of services. The use of HIV-focused funding to increase numbers in the overall health workforce is increasingly essential to scale up success.

The Mozambique experience also shows that integration efforts must consider the logic of the existing system, which is structured around defined levels of care and geographical units of administration. Mozambique's 10 provinces are the key organizational divisions through which PHC services are managed, coordinated and brought to scale. Transport, drug and material distribution, supervision, and data collection systems are organized administratively and logistically by the province and should be strengthened and harmonized at that level for integration to succeed. If limited to isolated sites or districts, integration will be ineffective and unsustainable if disconnected from provincial system strengthening. This approach contrasts with other vertical or NGO-led approaches that focus narrowly on single sites or small geographic areas.

## Conclusions

It is likely that major funding for HIV/AIDS services from large donors will continue to be channelled to "partners," such as NGOs, rather than to public sector health systems. Partners should coordinate closely with ministries of health to integrate HIV care into existing PHC services. This will necessarily mean a move away from vertical programming in smaller sites and adoption of a system-wide view that focuses support on appropriate MOH administrative divisions and processes. The rapid expansion of funding for HIV/AIDS programming provides a unique opportunity to improve all PHC services in African settings. The Mozambique experience so far shows that rapid ART scale up and system-wide strengthening must go hand in hand.

## Competing interests

The authors declare that they have no competing interests.

## Authors' contributions

JP conceived the project, supported project implementation, analyzed data, and wrote the manuscript. PM conceived the project, collected the data, supported project implementation, analyzed data, and wrote the manuscript. AJB conceived the project, supported project implementation, and helped draft the manuscript. MK conceived the project, supported project implementation, and helped draft the manuscript. MMP conceived the project, supported project implementation, and helped draft the manuscript. MM conceived the project, analyzed data, and helped draft the manuscript. KS conceived the project, supported project implementation, analyzed data, and helped draft the manuscript. SGS supported project implementation, analyzed data, and helped draft the manuscript. SB analyzed data and helped draft and edit the manuscript. BL analyzed data and helped draft and edit the manuscript. SG conceived the project, supported project implementation, analyzed data, and helped draft the manuscript. All authors read and approved the final manuscript.
